# Serine Carboxypeptidase SCPEP1 and Cathepsin A Play Complementary Roles in Regulation of Vasoconstriction via Inactivation of Endothelin-1

**DOI:** 10.1371/journal.pgen.1004146

**Published:** 2014-02-27

**Authors:** Xuefang Pan, Lubov Grigoryeva, Volkan Seyrantepe, Junzheng Peng, Katrin Kollmann, Johanne Tremblay, Julie L. Lavoie, Aleksander Hinek, Torben Lübke, Alexey V. Pshezhetsky

**Affiliations:** 1 Department of Medical Genetics, CHU Sainte-Justine Research Center, University of Montreal, Montreal, Canada; 2 Izmir Institute of Technology, Department of Molecular Biology and Genetics, Urla Izmir, Turkey; 3 CHUM Research Center and the Department of Kinesiology, University of Montreal, Montreal, Canada; 4 Department of Biochemistry, University Medical Center Hamburg-Eppendorf, Hamburg, Germany; 5 Cardiovascular Research Program, The Hospital for Sick Children, University of Toronto, Toronto, Canada; 6 Department of Chemistry, Biochemistry I, Bielefeld University, Bielefeld, Germany; 7 Department of Anatomy and Cell Biology, McGill University, Montreal, Canada; University of California San Diego, United States of America

## Abstract

The potent vasoconstrictor peptides, endothelin 1 (ET-1) and angiotensin II control adaptation of blood vessels to fluctuations of blood pressure. Previously we have shown that the circulating level of ET-1 is regulated through its proteolytic cleavage by secreted serine carboxypeptidase, cathepsin A (CathA). However, genetically-modified mouse expressing catalytically inactive CathA S190A mutant retained about 10–15% of the carboxypeptidase activity against ET-1 in its tissues suggesting a presence of parallel/redundant catabolic pathway(s). In the current work we provide direct evidence that the enzyme, which complements CathA action towards ET-1 is a retinoid-inducible lysosomal serine carboxypeptidase 1 (Scpep1), a CathA homolog with previously unknown biological function. We generated a mouse strain devoid of both CathA and Scpep1 activities (DD mice) and found that in response to high-salt diet and systemic injections of ET-1 these animals showed significantly increased blood pressure as compared to wild type mice or those with single deficiencies of CathA or Scpep1. We also found that the reactivity of mesenteric arteries from DD mice towards ET-1 was significantly higher than that for all other groups of mice. The DD mice had a reduced degradation rate of ET-1 in the blood whereas their cultured arterial vascular smooth muscle cells showed increased ET-1-dependent phosphorylation of myosin light chain 2. Together, our results define the biological role of mammalian serine carboxypeptidase Scpep1 and suggest that Scpep1 and CathA together participate in the control of ET-1 regulation of vascular tone and hemodynamics.

## Author Summary

Arterial blood pressure is regulated by small peptide hormones (vasoactive peptides) that cause contraction or relaxation of the arterial wall. The blood and tissue levels of these peptides are controlled by two mechanisms: through their synthesis and through their inactivation by the enzymes that are capable of cleaving them. Our results demonstrate that vasoactive peptide endothelin-1, is inactivated by two homologous enzymes, lysosomal serine carboxypeptidase, cathepsin A and lysosomal serine carboxypeptidase 1. We have developed a mutant strain of mice that do not produce both enzymes and found that these mice rapidly develop high blood pressure and show a reduced degradation rate of endothelin-1. We also found that endothelin-1 causes higher contraction of arteries from mutant than from normal mice or mice that are deficient only in one of the two enzymes. Our mouse model provides insight into the functional engagement of lysosomal serine carboxypeptidases in pathophysiology of hypertension and may become a tool to explore whether induction of these enzymes would have any therapeutic value.

## Introduction

Vascular resistance of the mammalian circulation system is tightly regulated by many endogenous agents that influence the blood volume, and diverse functions of endothelium, vascular smooth muscle and myocardium. When the balance of these agents is disturbed, persistent systemic hypertension develops. Short regulatory peptides, endothelin-1 (ET-1) and angiotensin II (AII) are recognized among the most potent vasoactive regulators. Through their interaction with cell surface receptors both peptides can modulate blood pressure by contracting arteries, or by induction or suppression of vascular wall remodelling.

ET-1 also has mitogenic effects on vascular endothelium and smooth muscle [1], stimulates the secretion of atrial natriuretic peptide ANP and aldosterone and inhibits the release of renin to counteract its effects [2]. The elevated ET-1 values have been previously observed in human vascular and cardiovascular disorders such as acute myocardial infarction, congestive heart failure, ischemia, atherosclerosis, hypercholestemia, systemic and pulmonary hypertension [3]. ET-1 deficient mice showed abnormal fetal development and haemodynamics [4], whereas the overexpression of human ET-1 in mice caused vascular remodelling and endothelial dysfunction [5], [6].

AII is another potent blood pressure-inducing and mitogenic peptide that belongs to the renin-angiotensin system. It is derived from the precursor, angiotensin I (AI) by angiotensin converting enzymes (ACE or ACE2). Inhibitors of AII receptors, as well as ACE inhibitors normalize the high blood pressure and decrease inward remodelling of arteries [7].

The bioavailability and potency of AII and ET-1 can be regulated through many factors such as alteration of receptor density and affinity, up- and down-regulation of peptide synthesis or release, enzymatic activation (ACE and ACE2 for AII, ECE and MMP-2 for ET-1 [8]), or degradation (neutral endopeptidase NEP for ET-1 [9]–[11]). Previously we have shown that circulating ET-1 is inactivated by lysosomal carboxypeptidase, cathepsin A (CathA) widely distributed in mammalian tissues (reviewed in [12]). The majority of CathA in the cell is found in the lysosome but significant pool of the enzyme is also present at the cell surface and secreted outside the cell [12]. *In vitro* CathA rapidly inactivates ET-1 by converting it into biologically inactive des-Trp21-endothelin-1 [13], [14]. CathA also hydrolyze the last residue of AI transferring it into angiotensin 1–9 (A1–9), which can be further converted into AII by ACE, but at much slower rate [15]–[17]. We reported that a gene-targeted mouse expressing enzymatically inactive CathA with a Ser190Ala mutation in the active site nucleophile [18] showed reduced degradation rate of ET-1 and significantly increased arterial blood pressure. At the same time, tissues of *CathA^S190A^* mice retained about 10–15% of the carboxypeptidase activity measured against ET-1 suggesting presence of parallel/redundant catabolic pathway(s) [18].

In the current work we tested whether the source of the complementary ET-1-degrading activity is a lysosomal serine carboxypeptidase 1 (Scpep1), a CathA homolog with previously unknown physiological function. Our results show that mice with a double CathA/Scpep1 deficiency (DD mice) demonstrate hypertension, increased ET-1-induced vasoconstriction and prolonged half-life of circulating ET-1 as compared to both wild type (WT) animals and those with single CathA or Scpep1 deficiencies, strongly supporting this hypothesis.

## Results

### Scpep1 has carboxypeptidase activity against ET-1

Mice with a combined deficiency of CathA and Scpep1 were obtained by intercrossing previously described *CathA^S190A^* and *Scpep1^−/−^* mouse lines, both in C57BL/6NCrl genetic backgrounds. Double heterozygous mice were crossed to obtain double homozygous *CathA^S190A^/Scpep1^−/−^* progeny, and their genotypes were confirmed by PCR of tail DNA (Fig. S1). *CathA^S190A^/Scpep1^−/−^* mice (double-deficient mice, DD) were viable and born in the frequency expected from Mendelian inheritance (16 of 307) indicating that combined deficiency of both enzymes does not cause embryonic lethality. *CathA^S190A^/Scpep1^−/−^* mice showed normal growth, were behaviourally indistinguishable from WT animals and could be bred to produce knockout litters.

**Figure 1:**
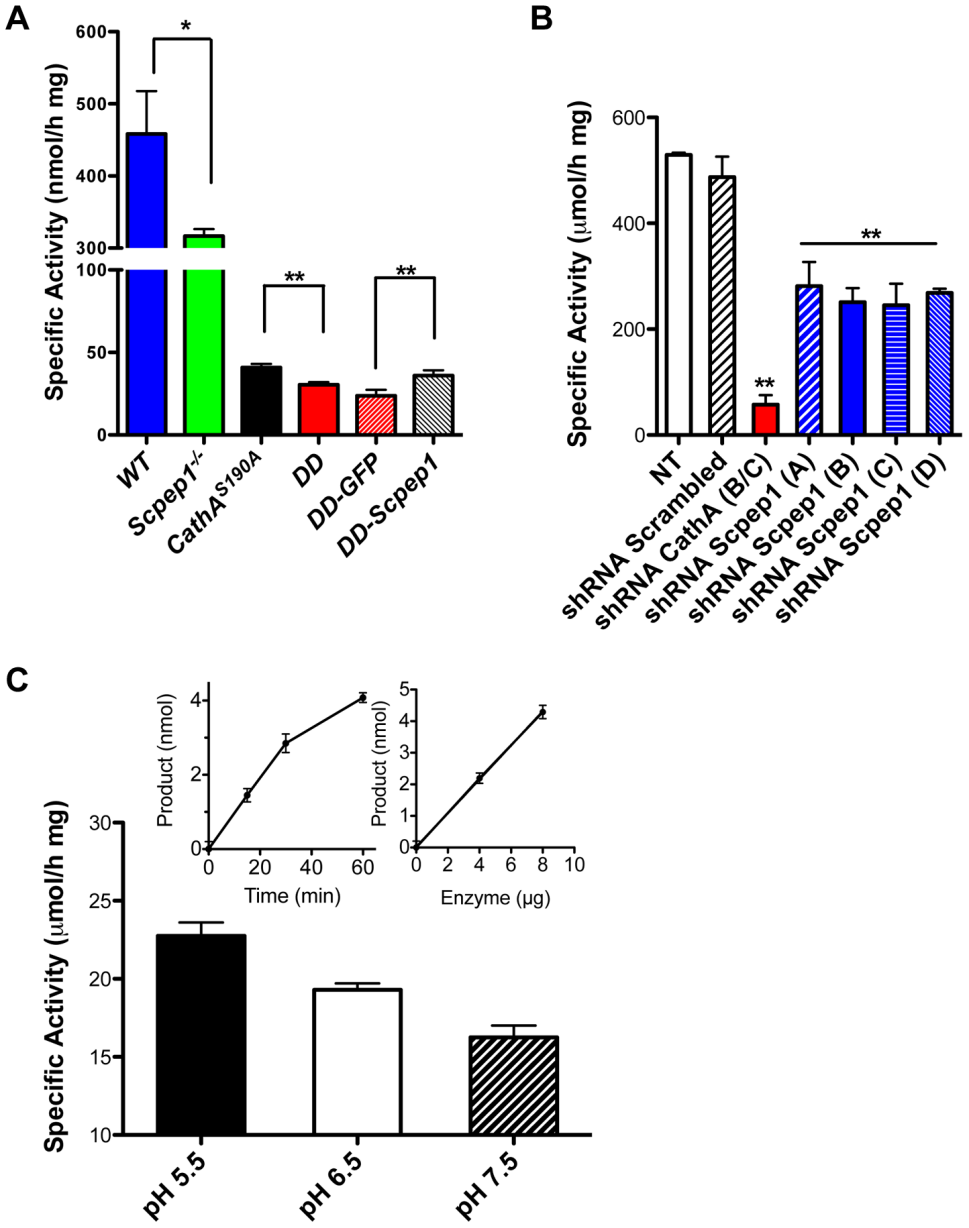
Scpep1 has carboxypeptidase activity against ET-1. A. Carboxypeptidase activity against ET-1 in homogenates of cultured AVSMC from WT, *Scpep1* ^−/−^, *CathA^S190A^* and *Scpep1* ^−/−^ */CathA^S190A^* mice as well as from the cells of *Scpep1* ^−/−^ */CathA^S190A^* mice transiently transfected with pCMV-GFP and pCMV-Scpep1 plasmids. AVSMC after the passage 3 were transfected or not with Scpep1-RGS-His-Tag and pEGFP-C1 plasmids. Forty-eight hours after transfection confluent cells were harvested, homogenized and tested for carboxypeptidase activity using 50 µM ET-1 as a substrate. Data are expressed as means (±S.D.) of 3 independent experiments performed with different cell cultures. * *p*<0.05 and ** *p*<0.01 in two-tailed unpaired t-test. B. Carboxypeptidase activity against ET-1 in homogenates of cultured AVSMC from WT mice transfected with Scpep1 and CathA shRNA. AVSMC after the passage 3 were transfected with pRFP-C-RS vectors expressing shRNA for mouse CathA and Scpep1 or non-effective 29-mer scrambled shRNA as indicated. Seventy-two hours after transfection the cells were harvested, and those expressing plasmids enriched by cell-sorting, homogenized and tested for carboxypeptidase activity using 50 µM ET-1 as a substrate. Data are expressed as means (±S.D.) of 3 independent experiments performed with different cell cultures. ** Significantly different from non-transfected or scrambled shRNA-transfected cells; *p*<0.01 in two-tailed unpaired t-test. C. Carboxypeptidase activity against ET-1 of purified recombinant Scpep1. Scpep1 carrying a His6 tag at the C-terminus was expressed in stably transfected HT1080 cells and purified by affinity chromatography on Ni-NTA resin followed by anion-exchange chromatography as described [19]. Carboxypeptidase activity was assayed with 50 µM ET-1 as a substrate and 0.4–0.8 µg of the purified enzyme in the reaction mixture in 50 mM sodium phosphate/50 mM sodium acetate buffer at pH 5.5, 6.5 and 7.5. Bars show mean values (±S.D.) of 3 independent experiments. In separate experiments we confirmed that under the conditions used the amount of liberated amino acid was directly proportional both to the incubation time and to the amount of Scpep1 protein in the reaction mixture (inset graphs). The amino acid analysis of the reaction mixture determined that Scpep1 cleaves the C-terminal amino acid (Trp21) from ET-1 (not shown).

The amount *Scpep1* mRNA measured by RT-q-PCR in aorta, hear and kidney tissues of *Scpep1^−/−^* and *CathA^S190A^/Scpep1^−/−^* mice (Fig. S2) was below detection limit. Carboxypeptidase activity against ET-1 assayed in cultured AVSMC of *CathA^S190A^* mice was reduced to ∼10% of activity in WT mice whereas the activity in tissues of *Scpep1^−/−^* mice was reduced to ∼70% (Fig. 1A). In the *CathA^S190A^/Scpep1^−/−^* mice carboxypeptidase activity was ∼6% of WT and significantly lower than that in *CathA^S190A^* mice, indicating that Scpep1 partially contributes to ET-1 hydrolysis (Fig. 1A). The activity of Scpep1 against ET-1 was further confirmed when the AVSMC of *CathA^S190A^/Scpep1^−/−^* mice were transiently transfected with Scpep1-expressing plasmid [19]. The level of carboxypeptidase activity measured with ET-1 in the transfected cells was significantly higher then in non-transfected cells or cells transfected with control plasmid, coding for green fluorescent protein (Fig. 1A) and similar to that in the cells of *CathA^S190A^* mice, despite the modest transfection level of ∼5% that could be achieved in the primary AVSMC cultures. In contrast when we transfected AVSMC from WT mice with a lentiviral vector expressing shRNA for Scpep1 the carboxypeptidase activity against ET-1 in the cell homogenate in the transfected cells was reduced by ∼50%, consistent with that in the AVSMC from *Scpep1^−/−^* mice. In the cells transfected with CathA shRNA-expressing vector the activity was decreased by ∼90% and in the cells transfected with scrambled RNA constructs, not changed (Fig. 1B).

Finally, to test directly if Scpep1 has carboxypeptidase activity against ET-1 we have expressed the protein, carrying a His6 tag at the C-terminus (Scpep1-His6) in HT1080 cells [19]. The secreted protein was purified until electrophoretic homogeneity by affinity chromatography on Ni-NTA resin followed by anion-exchange chromatography on Poros HQ resin (Fig. S3) and its carboxypeptidase activity was assayed as above with 50 µM ET-1 as a substrate. We found that at pH 5.5 purified Scpep1-His6 was capable of cleaving the C-terminal Trp residue from ET-1 at a rate of 23.6 µmol/h per mg of protein (Fig. 1C), i.e. close to that of purified CathA [20]. Lower activity was observed at higher pH of 6.5 and 7.5 (Fig. 1C).

### Scpep1 deficiency contributes to a further increase of blood pressure in CathA-deficient mice

The heart rate (HR) and blood pressure (BP) in WT, *CathA^S190A^*, *Scpep1^−/−^* and double-mutant *CathA^S190A^/Scpep1^−/−^* male mice was measured by radiotelemetry over a 3-day period. Then, the mice were challenged by a high salt diet (8% NaCl for 2 weeks) with continuous measurement of BP and HR.

**Figure 2:**
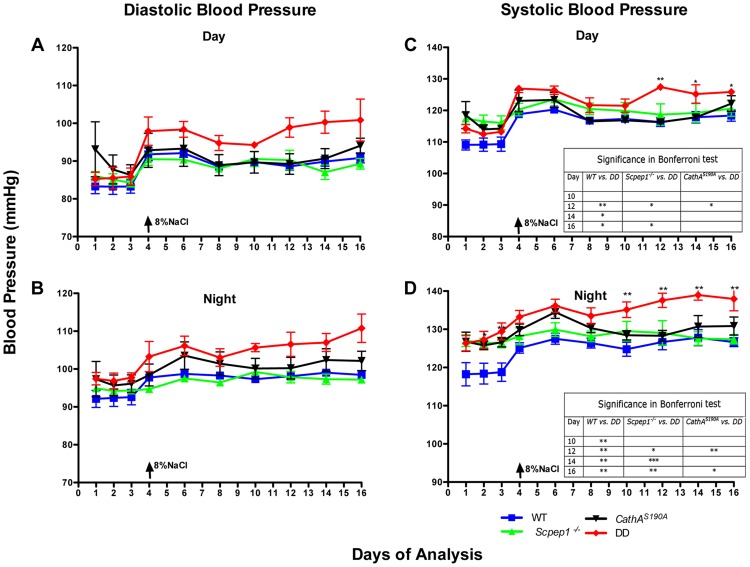
Mice with combined CathA/Scpep1 deficiency show significantly higher values of SBP. Blood pressure was recorded continuously during day (A and C) and night (B and D) 12-h periods in 16 week-old WT, *Scpep1* ^−/−^, *CathA^S190A^* and DD male mice. Arrows indicate commencement of high salt diet. Two-way repeated measurements ANOVA was used to test differences between the mouse groups: significant differences between the mean BP values in Bonferroni post-test (* *p*<0.05, ** *p*<0.001, *** *p*<0.0001) are shown in the insert. N-value for each genotype is as follows: WT n = 5, DD n = 6, *CathA^S190A^* n = 6, *Scpep1* ^−/−^ n = 7.

**Figure 3:**
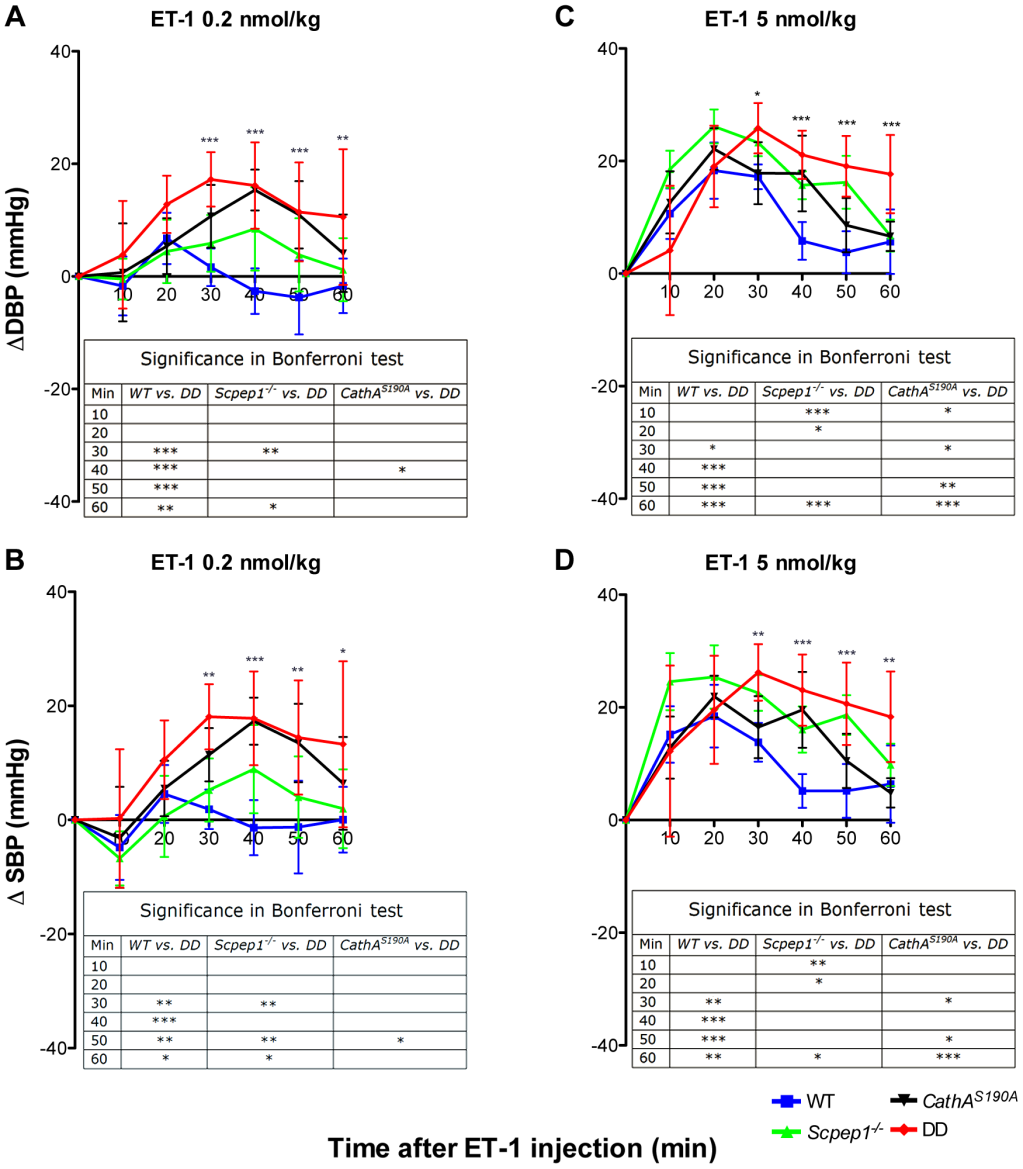
Mice with combined CathA/Scpep1 deficiency show higher increase in BP in response to systemic injection of ET-1. Sixteen week-old WT, *Scpep1^−/−^*, *CathA^S190A^* and DD male mice kept for two weeks on high-salt diet were intravenously injected with ET-1 solution in saline at a dose of 0.2 nmol/kg (A, B) and 5 nmol/kg (C, D). Changes in the systolic (ΔSBP) or diastolic (ΔDBP) blood pressure were calculated as differences between the means (±S.E) of the BP values recorded within 10 min intervals after the injections and means (±S.E) of the baseline BP values recorded within the 30 min interval before the injections. Two-way repeated measurements ANOVA was used to test differences between the mouse groups: significant differences between the mean BP values in Bonferroni post-test (* *p*<0.05, ** *p*<0.001, *** *p*<0.0001) are shown in the insert. N-value for each genotype is as follows: WT n = 5, DD n = 6, *CathA^S190A^* n = 6, *Scpep1* ^−/−^ n = 7.

The day (Fig. 2A, C) and night (Fig. 2B, D) levels of systolic BP (SBP) were significantly increased in *CathA^S190A^* animals as compared with WT both before and during a high-salt diet, whereas the BP levels in *Scpep1^−/−^* animals were similar to that of WT. Night SBP in *CathA^S190A^/Scpep1^−/−^* mice was significantly different from that of WT, Scpep1-deficient and CathA-deficient animals (Fig. 2 and Fig. S4). The HR values (Fig. S5) and the parameters characterizing kidney function (water intake, urine volume, urine sodium and urine creatinine levels, Fig. S6) were similar for all strains suggesting that the observed increase in SBP in DD mice relates to a vascular effect reflecting potential roles of Scpep1 and CathA in conversion of vasoconstrictive peptides. This hypothesis was further tested by measuring changes in BP in response to ET-1 and AI, the precursor of vasoconstrictive peptide AII. The changes in the diastolic and systolic BP (ΔDBP and ΔSBP, respectively) were calculated as the differences between the measured BP values and the basal values measured for the 30 min interval preceding the injection. To reduce the impact of the stress on BP caused by animal handling/injection, animals were receiving daily saline injections for 3 days prior to the experiment. The data (Fig. 3) indicate that the BP response to the i.v. injections of ET-1 was significantly (p<0.0001) dependent on animal genotype. The effect of ET-1 in Scpep-1-deficient mice was not significantly different from that in WT animals: there was no BP increase in response to the low (0.2 nmol/kg) dose of ET-1 and similar increase in response to the high (5 nmol/kg) dose. While CathA-deficient animals showed a higher response to ET-1 at the low dose as compared to WT or Scpep1-deficient animals only, DD animals showed a higher response as compared to all other groups of mice (Fig. 3A, B). After injection of ET-1 at the high dose the BP in DD mice remained significantly elevated for at least 60 min, whereas in WT or single knockout mice it decreased already after 40 min (Fig. 3C, D). The effect of AI on BP was similar in all animal groups (Fig. S7).

### Scpep1 deficiency increases the vasoconstrictive response of mesenteric arteries to ET-1 but not to AI

**Figure 4:**
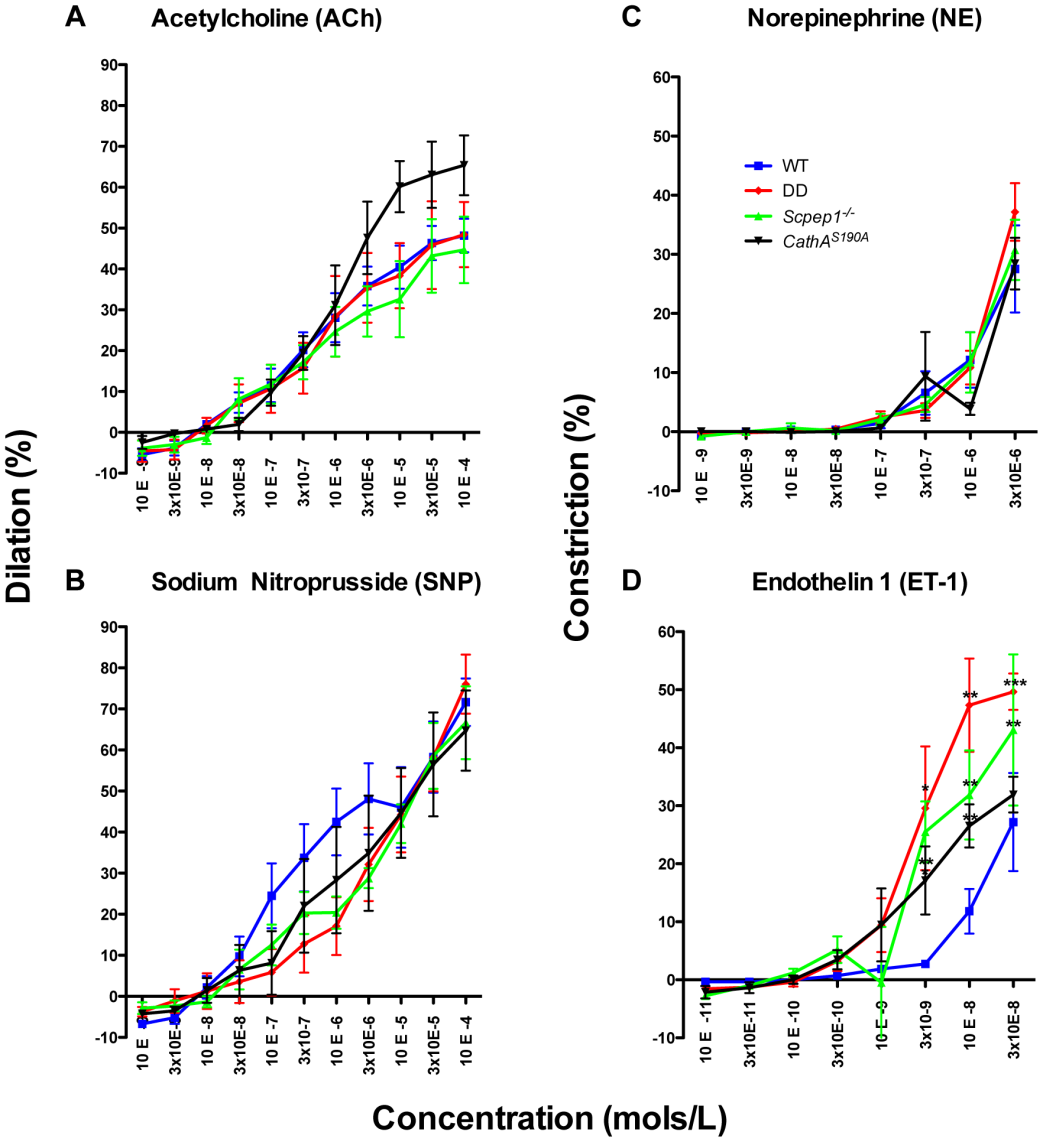
ET-1 causes significantly stronger constriction in mesenteric arteries from mice with Scpep1 deficiency. Mesenteric arteries isolated from sixteen week-old WT, *Scpep1^−/−^*, *CathA^S190A^* and DD male mice were treated with increasing concentrations of Ach (A), SNP (B) NE (C) and ET-1 (D). ET-1 caused significantly (p<0.01 two-way repeated measurements ANOVA) higher constriction of arteries from DD, *CathA^S190A^* and *Scpep1^−/−^* as compared to those from mice with other genotypes. Bonferroni post test: * *p*<0.05 ** *p*<0.01, *** *p*<0.001. N-value of each genotype is as follows: WT n = 8, DD n = 8, *CathA^S190A^* n = 6, *Scpep^−/−^* n = 6.

The vasoreactivity of mesenteric arteries from the four groups of male mice was directly measured in *ex-vivo* tests. Isolated arteries were exposed to ET-1 and the precursor of AII, AI as well as to known vasodilators (acetylcholine, ACh and sodium nitroprusside, SNP) and vasoconstrictors (norepinephrine, NE. We observed no differences in vessel reactivity in response to ACh, SNP or NE (Fig. 4A, B, C) as well as to AI (Fig. S8) between the four groups of mice. Reactivity to ET-1 was higher for *CathA^S190A^* and *Scpep1^−/−^* than for WT mice (Fig. 4D). The reactivity of vessels from *CathA^S190A^*/*Scpep1^−/−^* mice to ET-1 was significantly higher than that for all other groups of mice consistent with the *in vivo* data showing bigger increase of BP in *CathA^S190A^*/*Scpep1^−/−^* mice in response to ET-1 (Fig. 4D).

### Cultured AVSMC of CathA^S190A^/Scpep1^−/−^ mice show increased phosphorylation of myosin light chain

**Figure 5:**
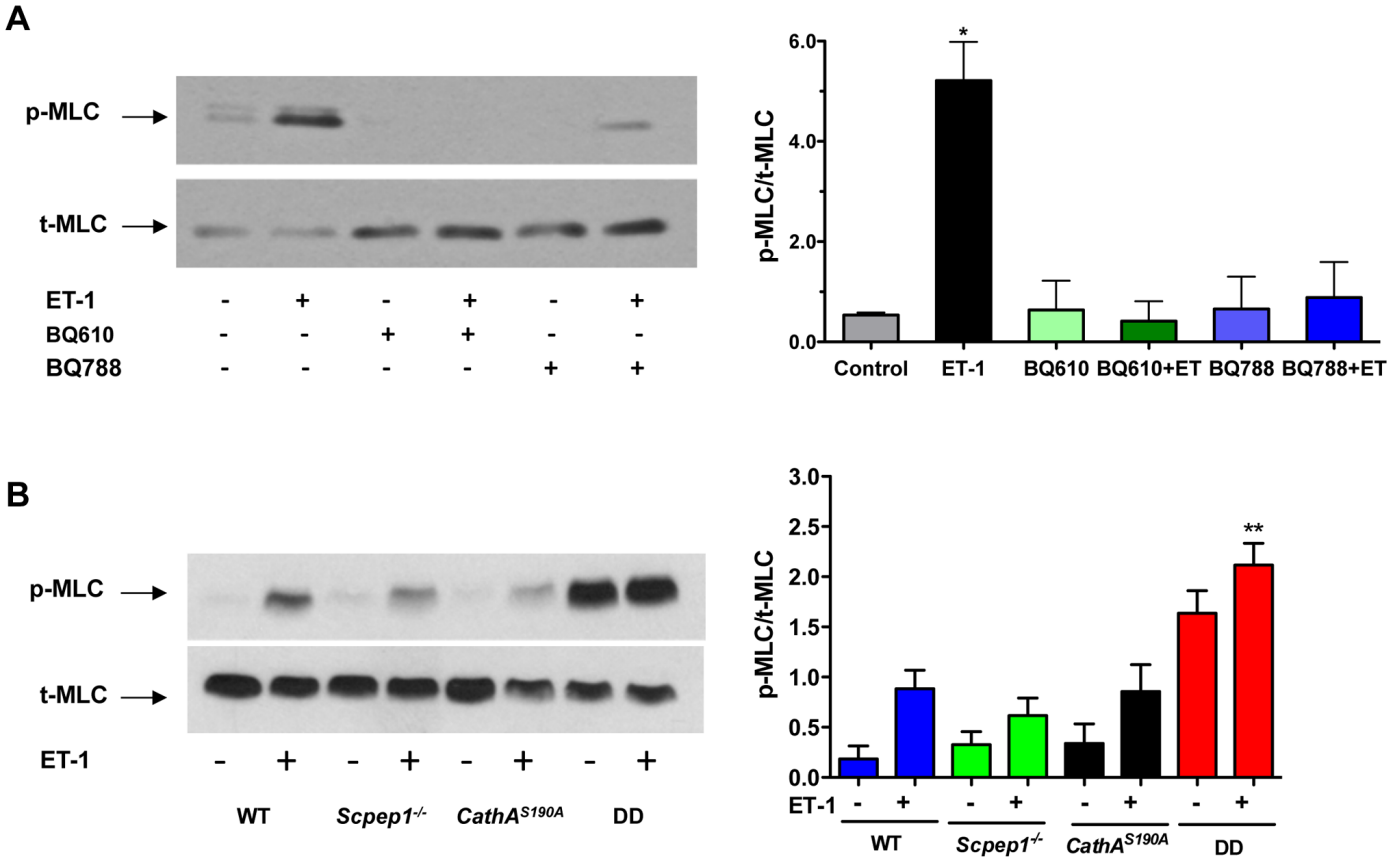
Cultured AVSMC from mice with combined CathA/Scpep1 deficiency show increased reactivity to ET-1. A. Pharmacological antagonists of ET receptors, BQ610 and BQ788, reduce MLC-2 phosphorylation in AVSMC treated with ET-1. Cultured AVSMC from WT mice were treated or not for 30 min with 2 µM BQ610 or BQ788 followed by 5 min induction with 100 nM ET-1 as indicated on the figure. Total protein extracts were analyzed by Western blotting using antibodies specific for phosphorylated (pThr^18^/Ser^19^-MLC) and total MLC-2 protein. Panel shows representative data of 3 independent experiments. Right panels show ratios (means and S.D.) of signal intensities for phosphorylated and total MLC-2 estimated with ImageQuant software. * *p*<0.05 in paired two-tailed *t*-test. B. Increased MLC-2 phosphorylation in AVSMC from double-deficient mice. Cultured AVSMC from WT, *Scpep1^−/−^*, *CathA^S190A^* and DD mice were treated for 5 min with 100 nM ET-1. Total protein extracts were analyzed by Western blotting using antibodies specific for phosphorylated and total MLC-2 protein. Panel shows representative data of 3 independent experiments. Graph below the panel shows ratios (mean values and S.D.) of signal intensities for phosphorylated and total MLC-2 protein. * *p*<0.05 in paired two-tailed *t*-test.

To verify at the molecular level if AVSMC from *CathA^S190A^*/*Scpep1^−/−^* mice have increased reactivity to ET-1, we studied intracellular signalling events in these cells in response to ET-1. ET-1 interacts with G-protein-associated endothelin type A (ETR-A) and type B (ETR-B) receptors on the surface of AVSMC. Activation of the receptors induces phospholipase C and increases the intracellular Ca^2+^ level leading to activation of myosin light chain kinase that phosphorylates myosin light chain (MLC) [21]–[24]. This causes contraction of myosin filaments and shrinkage of the cells. We therefore, compared the level of MLC phosphorylation in AVSMC before or after treatment with ET-1 for the 4 strains of mice. AVSMC cultured overnight in serum-free medium were treated with or without 100 nM ET-1, harvested and analyzed by Western blot using antibodies against MLC-2 phosphorylated at Thr^18^ and Ser^19^ residues or against total MLC protein. MLC-2 phosphorylation was blocked by pre-treatment of the cells with the known pharmacological antagonist of ETR-A, BQ610, and ETR-B antagonist, BQ788, suggesting that this effect is dependent on ET-1 action on its receptors (Fig. 5A). The cells from *CathA^S190A^*/*Scpep1^−/−^* mice had significantly higher level of pThr^18^/pSer^19^-MLC-2: ∼2 times higher than that in the control, CathA-deficient or Scpep1-deficient cells, and elevated basal levels of MLC-2 phosphorylation (Fig. 5B).

### CathA^S190A^/Scpep1^−/−^ mice demonstrate elevated levels of plasma ET-1

**Figure 6:**
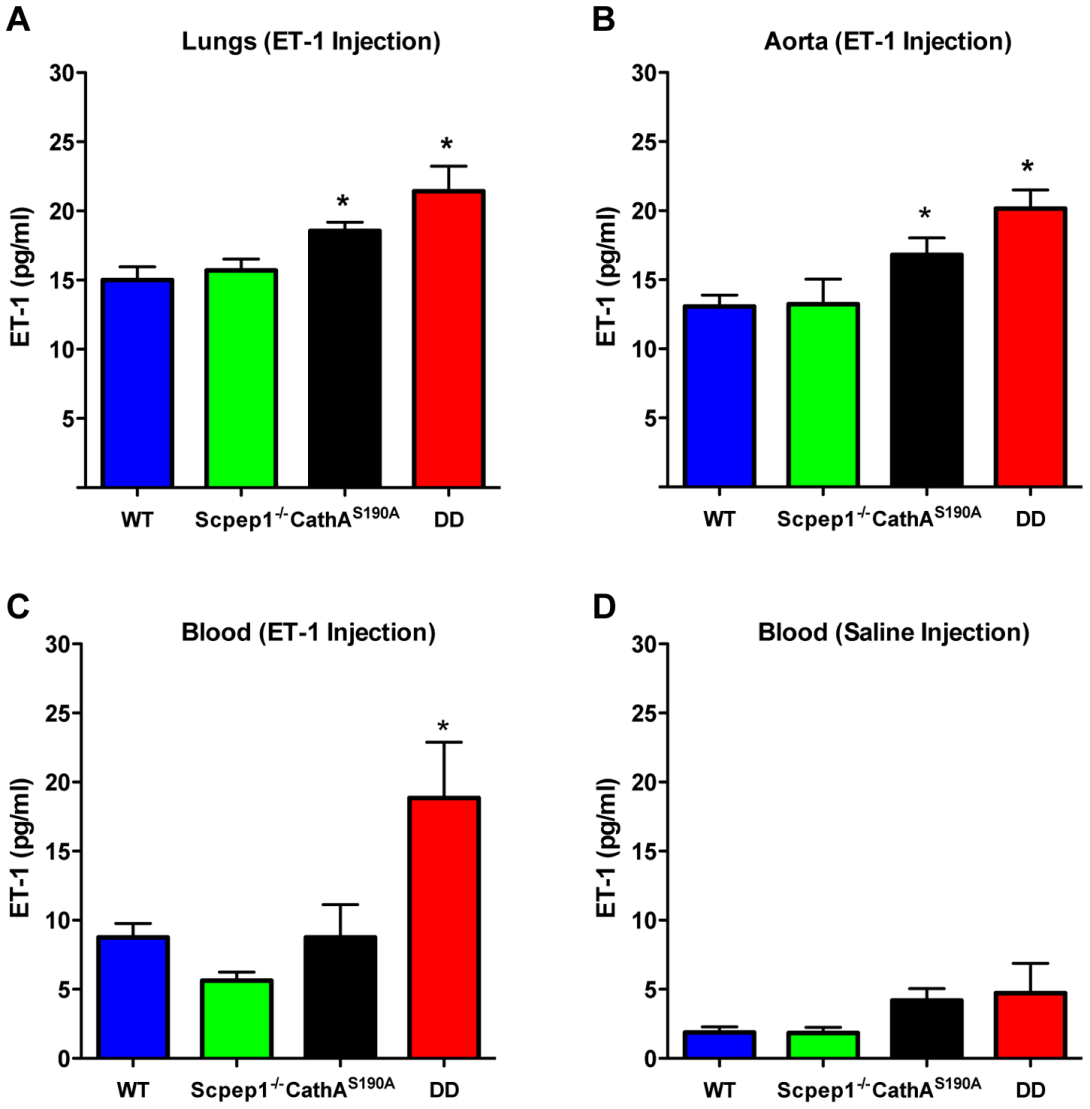
Mice with combined CathA/Scpep1 deficiency have reduced ET-1 degradation rate. Blood plasma, lungs and aorta were collected from WT, *Scpep1* ^−/−^, *CathA^S190A^* and DD mice 15 min post an intravenous injection of saline or ET-1 in saline (10 nmol/kg BW). The levels of ET-1 peptide were measured by ELISA in lung extracts (A), aorta extracts (B), and in blood plasma extracts (C) from mice injected with ET-1 and blood plasma extracts from mice injected with saline (D). Data are expressed as mean values (±S.E). At least 5 mice were studied for each genotype. * *p*<0.05 in Welch's modification of two-tailed unpaired t-test.

To determine the ET-1 degradation rate we injected mice in the tail vein with an ET-1 solution in saline at a dose of 0.1 nmol/kg BW. Fifteen minutes after injection mice were sacrificed and their lungs and aorta as well as blood were collected to measure the concentration of ET-1 by ELISA. Endogenous levels of ET-1 were measured in the animals injected with saline. Our data show that, 15 min after the ET-1 injection, its concentration in lungs (Fig. 6A) and aorta (Fig. 6B) of CathA-deficient mice was higher than that in the WT or Scpep1-deficient animals thus confirming our previous findings about the involvement of this enzyme in the ET-1 degradation. In tissues or plasma of DD mice the concentration of ET-1 was significantly higher than that in WT, CathA-deficient or Scpep1-deficient mice suggesting that in *CathA^S190A^/Scpep1^−/−^* mice the degradation rate of ET-1 is considerably reduced. No differences in endogenous circulating levels of ET-1 (Fig. 6D) were recorded.

## Discussion

Scpep1 was originally identified in rat aortic smooth muscle cells by screening for retinoid inducible genes [25]. Retinoids, natural and synthetic derivatives of vitamin A, block SMC proliferation and attenuate neointimal formation after vascular injury, presumably through retinoid receptor-mediated changes in gene expression. High transcript levels of Scpep1 were detected in kidney, lungs and heart. Scpep1 was localized to lysosomes by immunofluorescence, subcellular fractionation assays and mannose 6-phosphate receptor binding [19], [26].

Scpep1 shows high similarity to other members of the serine carboxypeptidase family and, in particular, to CathA. Like CathA, Scpep1 has a cleavable signal peptide, N-linked glycans, the Ser-Asp-His catalytic triad and is proteolytically processed from a 55 kDa precursor into the 35 kDa and 18 kDa fragments [19].

*Scpep1* gene-interrupted mice generated by us using a gene trap technology are fertile, have normal growth, normal clinical blood and urine parameters and did not have pathological changes in any tissue examined [19]. Later study by Lee at al. [27] reported that the *Scpep1*-null mice generated by replacing exons 1 and 2 of the Scpep1 gene with Neo cassette show a decrease in medial and intimal cell proliferation as well as in vessel remodelling following arterial injury. The same study also reported that a ∼50% knockdown of endogenous *Scpep1* in mouse ASMC line showed dramatic decrease in serum-stimulated growth. This study did not identify a physiological substrate of Scpep1, but the authors concluded that Scpep1 and CathA have distinct functions and “non-overlapping pools of substrates that function in cardiovascular homeostasis”.

Our current data, however, provide evidence that both carboxypeptidases catabolize at least one common substrate, ET-1. Mice devoid of both CathA and Scpep1 activities show significantly higher BP on both normal and high-salt diet or in response to systemic injections of ET-1 as compared to WT mice or those with single deficiencies of CathA or Scpep1. ET-1 also causes higher constriction of mesenteric arteries from DD mice. Since the effects of other tested vasodilators and vasoconstrictors are similar, these results are consistent with increased sensitivity of arterial smooth muscle to ET-1. Indeed, in cultured AVSMC from DD mice ET-1 caused significantly increased phosphorylation of MLC-2 as compared with the control, CathA-deficient or Scpep1-deficient cells. Finally, the degradation rate of ET-1 in the blood plasma or aorta and lung tissues was significantly reduced in DD as compared to WT, CathA-deficient or Scpep1-deficient mice.

The cardiovascular effects of AI and AII concentration in mouse plasma (S. Ernest, unpublished) were similar in WT, CathA-deficient, Scpep1-deficient and DD mice. This contradicts previously proposed role of CathA in the generation of AII from AI [15]–[17] and suggests that in general ET-1 and AII are controlled by different sets of proteases. We cannot exclude, however, that CathA still may participate in AII regulation in specific tissues, such as heart atrium, where the rate of AI conversion to A1–9 by CathA constitutes ∼25% of that to AII by ACE [17]

Our data indicate that in mouse tissues CathA is sufficient for inactivation of ET-1, which justifies the apparent absence of phenotype in our line of Scpep1 KO mice. In contrast, Scpep1 activity is unable to fully compensate for the loss in CathA activity in the knock-in CathA-deficient mice that show elevated blood pressure [18]. In the absence of CathA, the Scpep1 activity becomes essential for degradation of ET-1 as demonstrated by induced BP and contractility of arteries in DD as compared to singe CathA KI mice. Importantly, CathA has also other functions non-overlapping with those of Scpep1, such as activation of sialidase Neu1 in the lysosome [12], regulation of elastogenesis through its function in elastin-binding protein complex [28], [29] and inactivation of bradykinin [30]. Intravenous bolus injections of potent specific CathA inhibitors induced bradykinin-dependent diuresis [30], however in our experiments we did not see a difference in the urine volume between WT and CathA-deficient mice. One possible explanation is that CathA KI animals could adapt to deficiency of CathA by reducing bradykinin production or the number of bradykinin receptors.

The expression of *Scpep1* in cardiovascular tissues can be effectively induced by retinoic acid, potentially providing a metabolic bypath to correct arterial hypertension attributed to a deficiency in ET-1 degradation in galactosialidosis patients with mutations in the *CATHA* gene [31], [32]. Interestingly all-trans retinoic acid has been shown to inhibit pulmonary hypertension induced by monocrotaline in rats [33], whereas human patients with idiopathic pulmonary arterial hypertension were shown to have reduced retinoic acid levels [34]. The anti-hypertensive effect of retinoic acid treatment was attributed to its ability to elicit growth-inhibitory signals in pulmonary artery smooth muscle cells and influence pulmonary vascular remodelling [34]–[36], while our current data allow to propose that it may be also related to the induction of Scpep1 followed by increased degradation of ET-1. Together, our results define a biological role of Scpep1 protein, and suggest that Scpept1 and CathA participate together in the control of ET-1 regulation of vascular tone and hemodynamics.

## Methods

### Animals

Generation of mice containing Ser190Ala point mutation in the CathA active site (*CathA^S190A^* strain) and those with the *Scpep1* gene interrupted by gene-trap technology (*Scpep1^−/−^* strain) have been described [18], [19]. In the *Scpep1* gene-trap mouse β-galactosidase/neomycin phosphotransferase (*geo*) fusion gene was inserted into intron 7 of the *Scpep1* gene resulting in deletion of downstream exons 8–13 encoding in particular the putative catalytic triad amino acids, Asp371 and His431 from the gene trap transcript. The amount of *Scpep1* mRNA and protein measured by Northern and Western blots in liver, kidney, heart, brain spleen and lung tissues of *Scpep1^−/−^* mice [19] as well as the amount *Scpep1* mRNA measured by RT-q-PCR in aorta, hear and kidney tissues (Fig. S2) was reduced below detection threshold of the methods.

Both strains were back-crossed for at least 5 generations to C57BL/6NCrl strain distributed by Charles River (QC, Canada). Homozygous animals from each genotype were cross-bread to obtain the Scpep1-deficient, CathA-deficient, double-mutant and wild type mice. Mice were housed in an enriched environment with continuous access to food and water, under constant temperature and humidity, on a 12 h light∶dark cycle. Approval for the animal care and the use in the experiments was granted by the Animal Care and Use Committee of the Ste-Justine Hospital Research Center.

### Genotyping of mice

50 µl of PCR mixture contained 100 pmol of each primer, 0.2 mM dNTPs, 1.5 U taq polymerase (Feldan, 9K-001-0002) and 100 ng of genomic DNA from clipped tail tips in 20 mM Tris (pH 7.4), 50 mM KCl, and 1.5 mM MgCl_2_. Multiplex primers for detection of *Scpep1* alleles were 5′-ATCCTCACACATGCAAAGCA (Scpep1-F), 5′-TATTGGGCTGGAGTGGAGAC (Scpep1-R) and 5′- CCTGGCCTCCAGACAAGTAG (Scpep1-trap) and for detection of CathA alleles, 5′-GGTGGCGGAGAACAATTATG (CathA-F) and 5′-AACAGAAGTGGCACCCTGAC (CathA-R). For Scpep1 allele genotyping, samples were denatured at 94°C for 2 min, followed by 35 cycles at 94°C for 15 s, 52°C for 15 s and 72°C for 1 min, with a final extension reaction at 72°C for 30 s. For CathA allele genotyping, samples were denatured at 92°C for 5 min, followed by 30 cycles at 92°C for 30 s, 56°C for 30 s and 72°C for 30 s, with a final extension reaction at 72°C for 5 min. Then the amplification product was digested with *NdeI* (Biolabs, R0111S) at 37°C overnight.

### Quantitative RT-PCR

Total RNA was isolated from mouse tissues using the Trizol Reagent (Invitrogen 15596-026) according to the manufacturer's protocol and reverse-transcribed using random primers and QuantiTect Reverse Transcription Kit (QIAGEN 205311). Quantification of mouse *Scpep1* mRNA was performed using an SsoFast EvaGreen Supermix with Low ROX (BIO-RAD 172-5210) and the following set of primers: 5′- AGCAAGGGACCATTAAGTGC-3′ and 5′-GCTGAGTGGCCTCCTTGTAG-3′. PCR conditions were as follows: 30 sec at 95°C, followed by 40 cycles of 5 sec at 95°C, 20 sec at 60°C, and 20 sec at 72°C. RPL32 mRNA was used as a reference control; the data were expressed as signal ratios between the test gene mRNA and RPL32 mRNA.

### Blood pressure measurements by radiotelemetry

Male *CathA^S190A^* mice and appropriate littermate controls were implanted with TA11PA-C10 radiotelemetry sensors (Data Sciences International) in the left carotid artery for direct measurement of arterial pressure and heart rate as described [37], [38]. The transmitter was placed subcutaneously along the left flank. For basal measurements of mean day and night BP data were recorded continuously (sampling every hour for 20 sec) within 16 consecutive days and averaged for 12 h light and dark intervals. To measure changes in BP after ET-1 and AI injections data were recorded every 3 min for 2 h and averaged for 10-min consecutive intervals. At least 7 mice were studied for each genotype with the exception of WT mice for which only 5 mice were tested due to sudden death of 2 animals.

### Vessel reactivity study

Vessel reactivity *ex vivo* was analyzed as described [39], [40]. Briefly, male mice were sacrificed at five months and their mesenteric arteries were isolated and mounted onto glass capillaries in an artereograph chamber filled with cold oxygenated Krebs solution (118.6 mM NaCl, 4.7 mM KCl, 1.2 mM KH_2_PO_4_, 1.2 mM MgSO_4_, 25.1 mM NaHCO_3_, 26 µM EDTA, 0.18% glucose, 2.5 mM CaCl_2_). The arteries were constantly perfused intraluminally with Krebs solution at 30 mmHg. After 45 minutes of equilibration vascular reactivity was measured in response to Norepinephrine (Sigma A-0937, 10^−9^–10^−5^ M), Acetylcholine (Sigma A-6625, 10^−9^–10^−4^ M), Sodium Nitroprusside (Calbiochem 56538, 10^−9^–10^−4^ M), Endothelin-1 (American Peptide Company 88-1-10, 10^−11^–10^−8^ M) and AI, (American Peptide Company 12-1-10, 10^−8^–10^−4^ M). Drugs were added extraluminally with a 30 min washout period in between each drug, during which the arteries were able to re-equilibrate to a baseline. To test vasodilatation arteries were pre-contracted with NE to 70% of their equilibration diameter. At least 3 concentration response curves were conducted for each vessel and at least 6 animals were studied for each genotype.

### Isolation and culture of aortic vascular smooth muscle cells (AVSMC)

Combined tissues from 5–6 mouse aortas were minced in a DMEM containing collagenase type I (GIBCO, 17100-017, 3 mg/ml), trypsin (Sigma T-1426, 0.5 mg/ml), and DNAse type I (Sigma D-4263, 20 µg/ml), incubated at 37°C for 2 h, and centrifuged for 5 min at 1000 g and 4°C. The cells were resuspended in 10 ml of DMEM containing 10%FBS, 1% Antibiotic-Antimycotic (GIBCO 15240-062), 0.5% Fungizone (GIBCO 15290-018) and maintained in 5% CO_2_ incubator at 37°C. The medium was changed every three days. After 3 passages 100% of cells were positive to VSMC marker, smooth muscle α-actin as assayed by FACS with A 2547 antibody (Sigma).

### Purification of recombinant mouse Scpep1-His6 from HT1080 cells

HT1080 cells stably expressing Scpep1-His6 [19] were cultured in DMEM with 0.05% FCS. Medium was collected three times every 48 h and subjected to ammonium sulfate precipitation. After dialysis against PBS, the Scpep1-His6 was purified by metal affinity chromatography on Ni-NTA agarose (Qiagen) as recommended by manufacturer. The eluate was dialyzed against PBS and subjected to HPLC anion exchange chromatography (BiocadVision, Applied Biosystems) by applying a step-wise gradient up to 500 mM NaCl in PBS. Purity of Scpep1-His6 was monitored by silver staining and Western blotting.

### Carboxypeptidase activity assays

Carboxypeptidase activity in cultured AVSMC was measured against 50 µM ET-1 as previously described using the method measuring the liberation rate of the C-terminal amino acid of the peptide [18]. Subconfluent AVSMC were transiently transfected or not with Scpep1-RGS-His-Tag [19] and pEGFP-C1 (Clontech, Palo Alto, CA) plasmids, mouse CTSA shRNA (TF501716B/C) Scpep1 shRNA (TF505007A/B) or non-effective 29-mer scrambled shRNA (TR30015) cassette in pRFP-C-RS vector (Origene Technologies) using Effectene transfection reagent (Qiagen) at a ratio of 25 µl of Effectene to 1 µg of DNA. Forty eight hours after transfection (72 h for shRNA constructs) confluent cells were harvested, homogenized in water by sonication and 50 µl of cell homogenate was mixed with 100 µl of 0.1 mM ET-1 solution and 50 µl of 100 mM sodium acetate buffer, pH 5.4, and incubated for 30–180 min at 37°C. After addition of trichloroacetic acid (Sigma T0699, 3% final concentration) proteins were removed by 5 min centrifugation at 12,000 g. The 190 µl aliquot of supernatant was mixed with 3 ml of 50 mM sodium borate buffer, pH 9.5, containing 0.15 mg/ml of phthalic aldehyde and 1 mM of beta-mercaptoethanol (Sigma, M-6250) and incubated at room temperature for 30 min. The fluorescence was measured at 340 nm excitation and 495 emission wavelength and concentration of released amino acids determined using a calibration curve established with 1–100 µM leucine. Carboxypeptidase activity of recombinant Scpep1-His6 was measured by the same method using 0.4–0.8 µg of the purified enzyme.

### Analysis of myosin light chain 2 phosphorylation by western blot

AVSMC cultured in 100 mm dishes to confluent layer were incubated overnight in a serum-free DMEM, and treated for 5 min with 100 nM ET-1. To test the pharmacological inhibition of the ET-1 receptors the cells were pre-treated for 30 min with 2 µM BQ610 (EMD 203715) or BQ788 (EMD 5223838) before stimulation with ET-1. The cells were washed with ice-cold PBS, and lysed in RIPA (RadioImmunoPrecipitation Assay) buffer containing 50 mM Tris HCl, pH 7.4, 150 mM NaCl, 1% NP-40, 0.25% sodium deoxycholate, 0.1% SDS, 2 mM EDTA, 1 mM PMSF, protease and phosphatase inhibitor cocktails (Roche 04693132001 and 04906837001). Cell lysates were analyzed by Western blot using anti-phospho-Thr^18^/Ser^19^ myosin light chain 2 antibody (Cell Signalling 3674, dilution 1∶1000) or anti-myosin light chain 2 antibodies (Cell Signalling 3672, dilution 1∶1000). Detection was performed with anti-rabbit IgG antibodies-HRP conjugate (Cell Signalling 7074S), and the enhanced chemiluminescence reagent (Thermo 32106).

### Measurement of ET-1 degradation rate in mouse blood and tissues

Three to four month old mice with 25–35 g body weight (BW) were anesthetised with urethane (1.5 g/kg BW) and injected into the tail vein with a solution of ET-1 in saline at a dose of 10 nmol/kg BW. Fifteen minutes post-injection, blood was collected in EDTA-coated tubes through cardiac puncture and immediately centrifuged to separate plasma. Aortas and lungs were dissected and rapidly frozen in liquid nitrogen.

For peptide extraction, tissues (200 mg) were homogenized in 1 mol/L CH_3_COOH/20 mM HCl. Plasma was supplemented with concentrated CH_3_COOH until the final concentration of 1 mol/L. Samples were boiled for 10 minutes and centrifuged at 20,000 *g* for 10 minutes. Supernatant was applied to a Strata C18-E column (Phenomenex, RK-Sepcol-1), washed with 3 volumes of 0.1% TFA in water, and peptides were eluted with 60% acetonitrile/0.1% TFA, lyophilized, and reconstituted in 0.1% TFA in DMSO. Quantitative assay of ET-1 was performed with an ELISA kit (Enzo Life Sciences ADI-900-020A) as described by the manufacturer.

### Statistical analysis

Statistical analysis has been performed using two-tailed paired t-test (Fig. 1, and 4), Welch's modification of two-tailed unpaired t-test (Fig. 5, S2 and S8) and two-way repeated measures ANOVA (Fig. 2, 3, and 4) tests using Prism Graphpad software. P-value of 0.05 or less was considered significant. Bonferroni post-hoc test was used to compare specific means, if significance was determined.

### Statement of responsibility

The authors had full access to the data and take responsibility for its integrity. All authors have read and agreed to the manuscript as written.

## Supporting Information

Figure S1 Genotyping of WT, *Scpep1^−/−^*, *CathA^S190A^* and double deficient (DD) mice by PCR analysis of tail genomic DNA. (A) *Scpep1* allele-specific PCR amplifying a 200 bp fragment in wild type (WT) mice and 390 bp fragment in homozygous *Scpep1*-deficient animals (*Scpep1^−/−^*). (B) *CathA* allele-specific PCR followed by *NdeI* digestion produces a 350 bp fragment in wild type (WT) mice, and a 250 and 100 bp fragments in homozygous *CathA*-deficient animals (*CathA^S190A^*).
(PDF)

Figure S2 *Scpep1* mRNA expression in mouse tissues. (A) Schematic representation of WT and *Scpep1* ^−/−^ mRNA showing the positions of primers for qPCR in Exon 6 and Exon 8. (B) *Scpep1* relative mRNA expression in heart, kidney and aorta tissues. Total RNA was extracted from tissues of 16 week-old WT, *Scpep1* ^−/−^, *CathA^S190A^* and double-deficient (DD) mice and analyzed for *Scpep1* expression in different tissues by qPCR. The values were corrected for the level of control RPL32 mRNA.
(PDF)

Figure S3 Purification of recombinant mouse Scpep1-His6 from stably expressing HT1080 cells. Scpep1-His6 expressing HT1080 cells (Kollmann et al. 2009, FEBS Journal) were cultured in 0.05% FCS in DMEM. Medium was collected three times every 48 h and subjected to ammonium sulfate precipitation. After dialysis to PBS, the Scpep1-His6 was purified by Ni-NTA agarose (Qiagen). The eluate was dialyzed to PBS and subjected to HPLC anion exchange chromatography (BiocadVision, Applied Biosystems) by applying a step-wise gradient up to 500 mM NaCl in PBS. Purification was monitored by silver staining and Western blotting. Fractions 9 and 10 were pooled and used for the assay of carboxypeptidase activity.
(PDF)

Figure S4 Mice with combined CathA/Scpep1 deficiency show significantly higher values of SBP. Diastolic (A) and systolic (B) blood pressure was recorded continuously (1 measurement per hour) during day and night 12-h periods in 16 week-old WT, *Scpep1* ^−/−^, *CathA^S190A^* and DD male mice. Arrows indicate commencement of high salt diet. Two-way repeated measurements ANOVA was used to test differences between the mouse groups: significant differences between the mean BP values in Bonferroni post-test (* *p*<0.05, ** *p*<0.001, *** *p*<0.0001) are shown in the insert. N-value for each genotype is as follows: WT n = 5, DD n = 6, *CathA^S190A^* n = 6, *Scpep1* ^−/−^ n = 7.
(PDF)

Figure S5 No significant differences were observed between heart rate in WT, *Scpep1^−/−^*, *CathA^S190A^* and double-deficient (DD) mice. Heart rate was recorded continuously (once each hour) during night (A) and day (B) 12-h periods in 16 week-old WT, *Scpep1* ^−/−^, *CathA^S190A^* and double-deficient (DD) mice fed for three days with normal diet, followed by two weeks on high salt diet. Arrows indicate commencement of high salt diet. Two-way ANOVA was used to test differences between the mouse groups. N-value for each genotype is as follows: WT n = 5, DD n = 6, *CathA^S190A^* n = 6, *Scpep1* ^−/−^ n = 7.
(PDF)

Figure S6 No significant differences in kidney function were observed between WT, *Scpep1^−/−^*, *CathA^S190A^* and double-deficient (DD) mice. Twenty four hour water intake was measured and urine collections obtained from 16 week-old male WT, *Scpep1* ^−/−^, *CathA^S190A^* and double-deficient (DD) mice fed with normal diet, or following two weeks on a high salt diet. Graphs present 24 h water intake (A) urine sodium (B), urine volume (C) and urine creatinine (D) measured as previously described. Values are shown as means (±S.E). N-value for each genotype is as follows: WT n = 5, DD n = 6, *CathA^S190A^* n = 6, *Scpep1* ^−/−^ n = 7.
(PDF)

Figure S7 No significant difference was observed in diastolic (A, B) and systolic (C, D) blood pressure in response to systemic injections of AI. Sixteen week-old WT, *Scpep1^−/−^*, *CathA^S190A^* and double-deficient (DD) mice kept for two weeks on a high salt diet were intravenously injected with AI solution in saline (0.08 and 0.8 nmol/kg BW) or saline only. The pressure was recorded continuously every 2 min for 30 min before and one hour after injections. Changes in the blood pressure (ΔSP or ΔDP) were calculated as differences between the BP values recorded within 10 min intervals after the injections and the baseline BP values recorded within the 30 min interval before the injections. Two-way repeated measurements ANOVA was used to test differences between the mouse groups. N-value of each genotype is as follows: WT n = 5, DD n = 6, *CathA^S190A^* n = 6, *Scpep1* ^−/−^ n = 7.
(PDF)

Figure S8 No significant difference was observed in the AI induced constriction of mesenteric arteries between *CathA^S190A^*, double deficient (DD) and WT mice. Mesenteric arteries isolated from sixteen week-old male WT, *Scpep1* ^−/−^, *CathA^S190A^* and double deficient mice were mounted onto glass capillaries in an artereograph chamber filled with cold oxygenated Krebs solution and treated with increasing concentrations of AI. Two-way repeated measurements ANOVA was used to test differences between the mouse groups. N-value of each genotype is as follows: WT n = 8, DD n = 8, *CathA^S190A^* n = 6, *Scpep1* ^−/−^ n = 6.
(PDF)
