# Supplementary figures and images for: Serine Carboxypeptidase SCPEP1 and Cathepsin A Play Complementary Roles in Regulation of Vasoconstriction via Inactivation of Endothelin-1
Source: PLoS Genet. 2014 Feb 27;10(2):e1004146. doi: 10.1371/journal.pgen.1004146 (PMC3937211; doi:10.1371/journal.pgen.1004146)

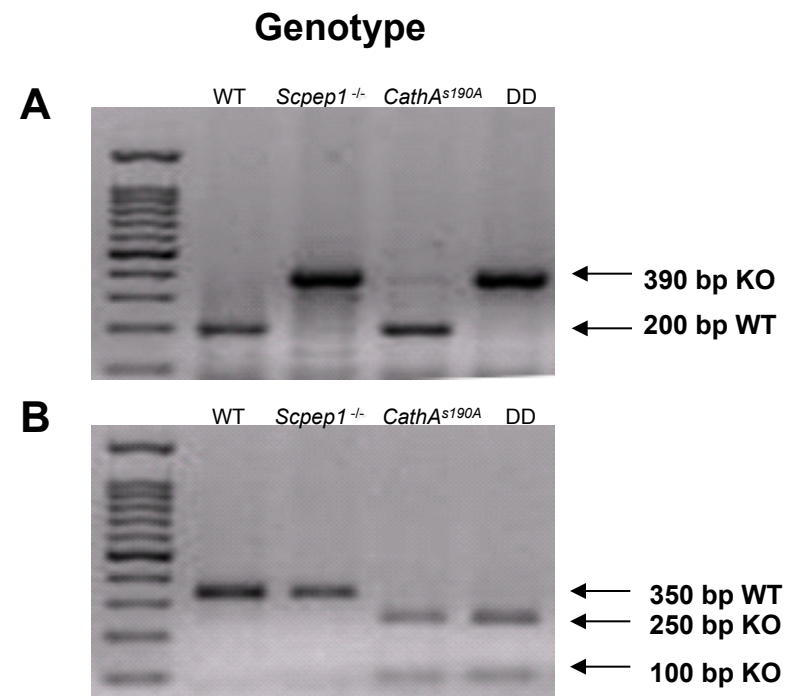

Supplementary figure 1

Supplement: Figure S1 — Genotyping of WT, Scpep1−/−, CathAS190A and double deficient (DD) mice by PCR analysis of tail genomic DNA. (A) Scpep1 allele-specific PCR amplifying a 200 bp fragment in wild type (WT) mice and 390 bp fragment in homozygous Scpep1-deficient animals (Scpep1−/−). (B) CathA allele-specific PCR followed by NdeI digestion produces a 350 bp fragment in wild type (WT) mice, and a 250 and 100 bp fragments in homozygous CathA-deficient animals (CathAS190A). (PDF) [file pgen.1004146.s001.pdf]

A

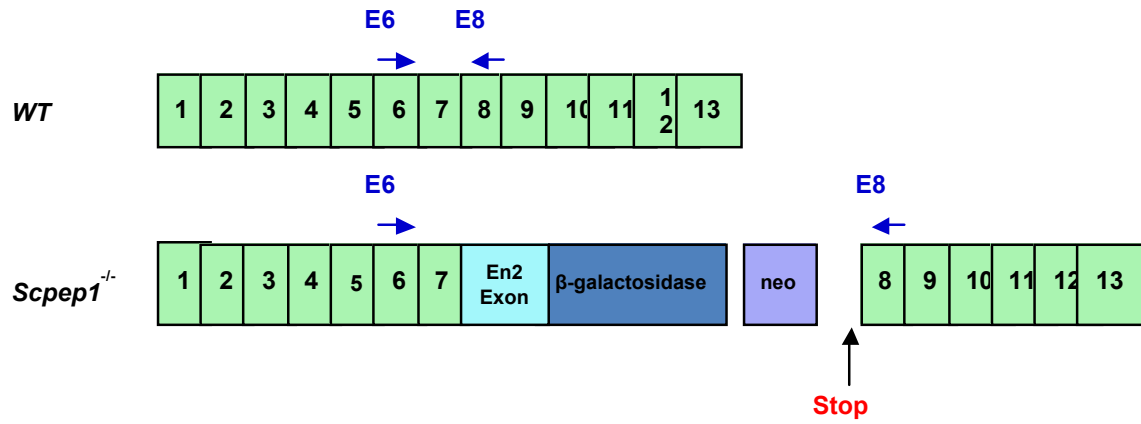

B

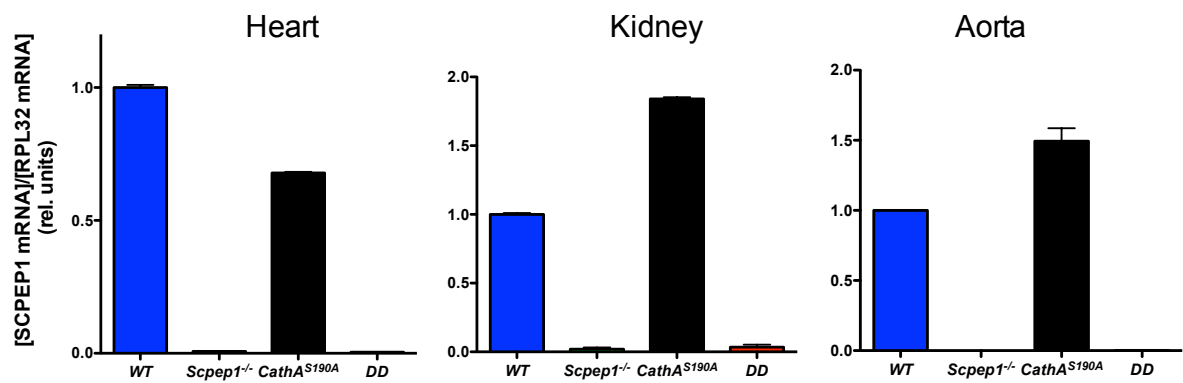

Supplementary figure 2

Supplement: Figure S2 — Scpep1 mRNA expression in mouse tissues. (A) Schematic representation of WT and Scpep1 −/− mRNA showing the positions of primers for qPCR in Exon 6 and Exon 8. (B) Scpep1 relative mRNA expression in heart, kidney and aorta tissues. Total RNA was extracted from tissues of 16 week-old WT, Scpep1 −/−, CathAS190A and double-deficient (DD) mice and analyzed for Scpep1 expression in different tissues by qPCR. The values were corrected for the level of control RPL32 mRNA. (PDF) [file pgen.1004146.s002.pdf]

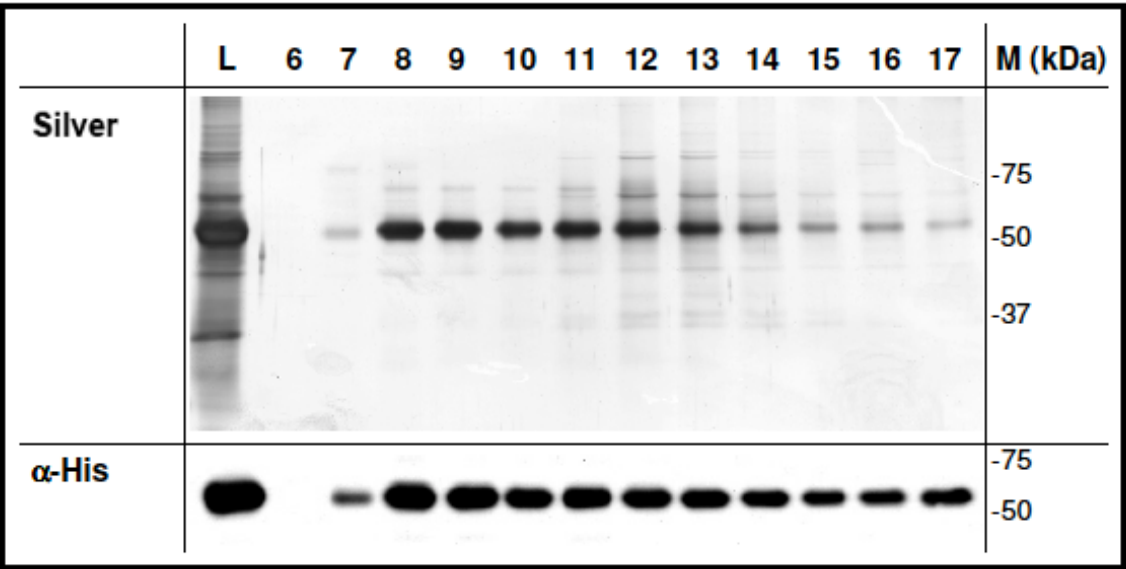

Supplementary figure 3

Supplement: Figure S3 — Purification of recombinant mouse Scpep1-His6 from stably expressing HT1080 cells. Scpep1-His6 expressing HT1080 cells (Kollmann et al. 2009, FEBS Journal) were cultured in 0.05% FCS in DMEM. Medium was collected three times every 48 h and subjected to ammonium sulfate precipitation. After dialysis to PBS, the Scpep1-His6 was purified by Ni-NTA agarose (Qiagen). The eluate was dialyzed to PBS and subjected to HPLC anion exchange chromatography (BiocadVision, Applied Biosystems) by applying a step-wise gradient up to 500 mM NaCl in PBS. Purification was monitored by silver staining and Western blotting. Fractions 9 and 10 were pooled and used for the assay of carboxypeptidase activity. (PDF) [file pgen.1004146.s003.pdf]

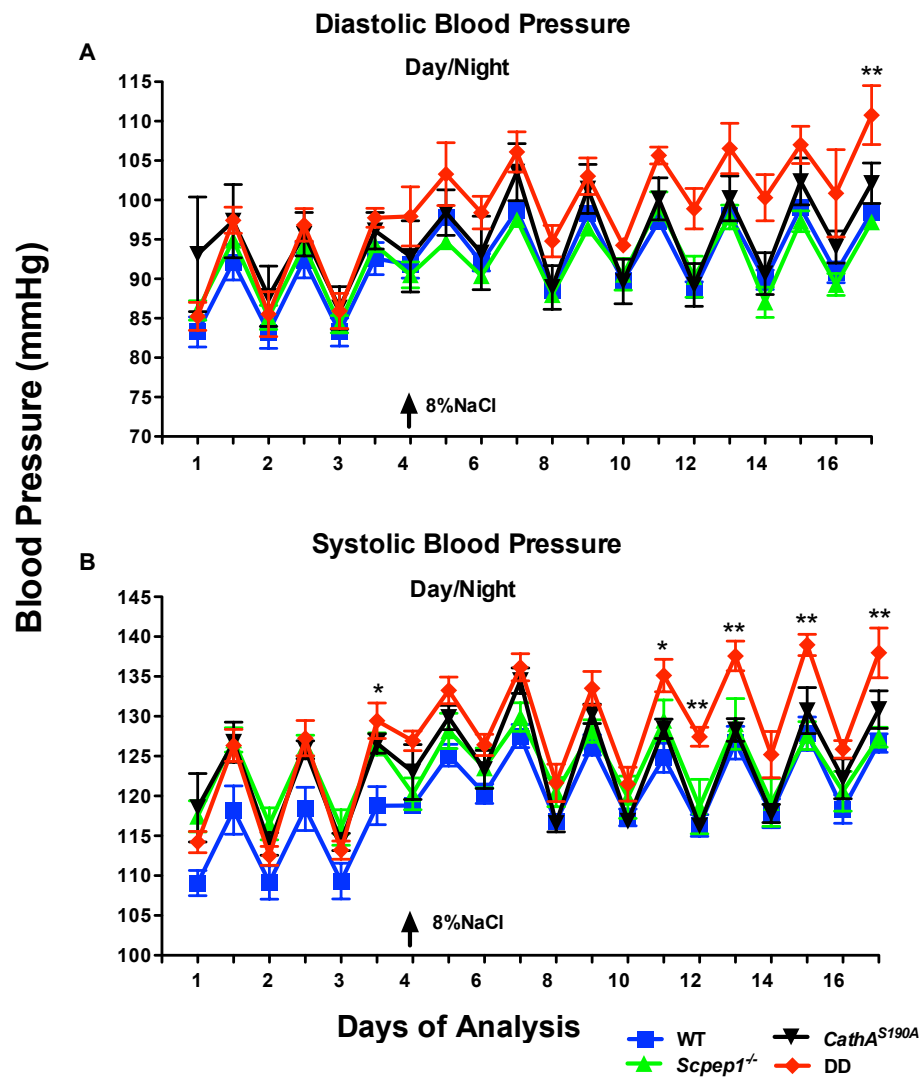

Supplementary figure 4

Supplement: Figure S4 — Mice with combined CathA/Scpep1 deficiency show significantly higher values of SBP. Diastolic (A) and systolic (B) blood pressure was recorded continuously (1 measurement per hour) during day and night 12-h periods in 16 week-old WT, Scpep1 −/−, CathAS190A and DD male mice. Arrows indicate commencement of high salt diet. Two-way repeated measurements ANOVA was used to test differences between the mouse groups: significant differences between the mean BP values in Bonferroni post-test (* p<0.05, ** p<0.001, *** p<0.0001) are shown in the insert. N-value for each genotype is as follows: WT n = 5, DD n = 6, CathAS190A n = 6, Scpep1 −/− n = 7. (PDF) [file pgen.1004146.s004.pdf]

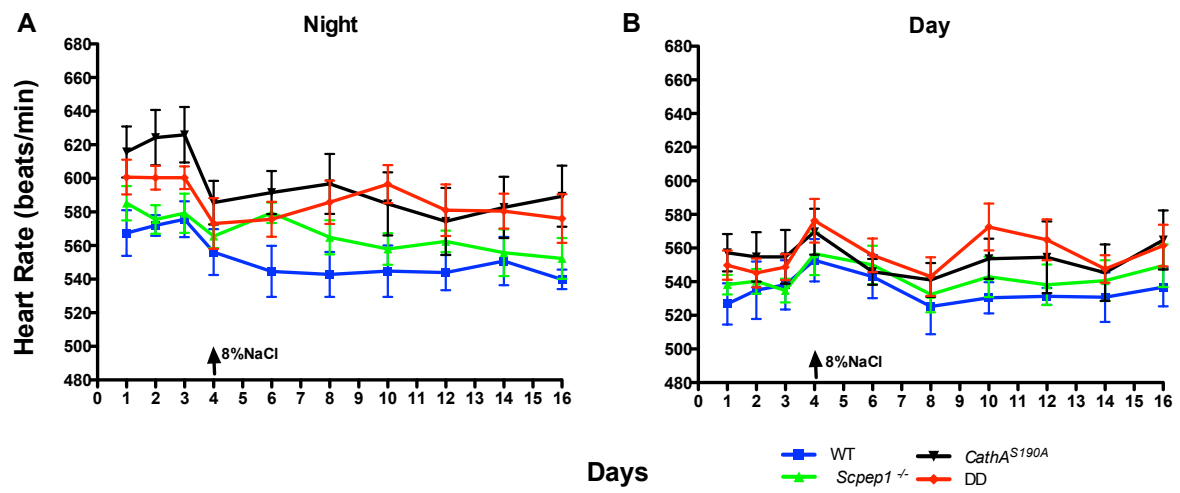

Supplementary figure 5

Supplement: Figure S5 — No significant differences were observed between heart rate in WT, Scpep1−/−, CathAS190A and double-deficient (DD) mice. Heart rate was recorded continuously (once each hour) during night (A) and day (B) 12-h periods in 16 week-old WT, Scpep1 −/−, CathAS190A and double-deficient (DD) mice fed for three days with normal diet, followed by two weeks on high salt diet. Arrows indicate commencement of high salt diet. Two-way ANOVA was used to test differences between the mouse groups. N-value for each genotype is as follows: WT n = 5, DD n = 6, CathAS190A n = 6, Scpep1 −/− n = 7. (PDF) [file pgen.1004146.s005.pdf]

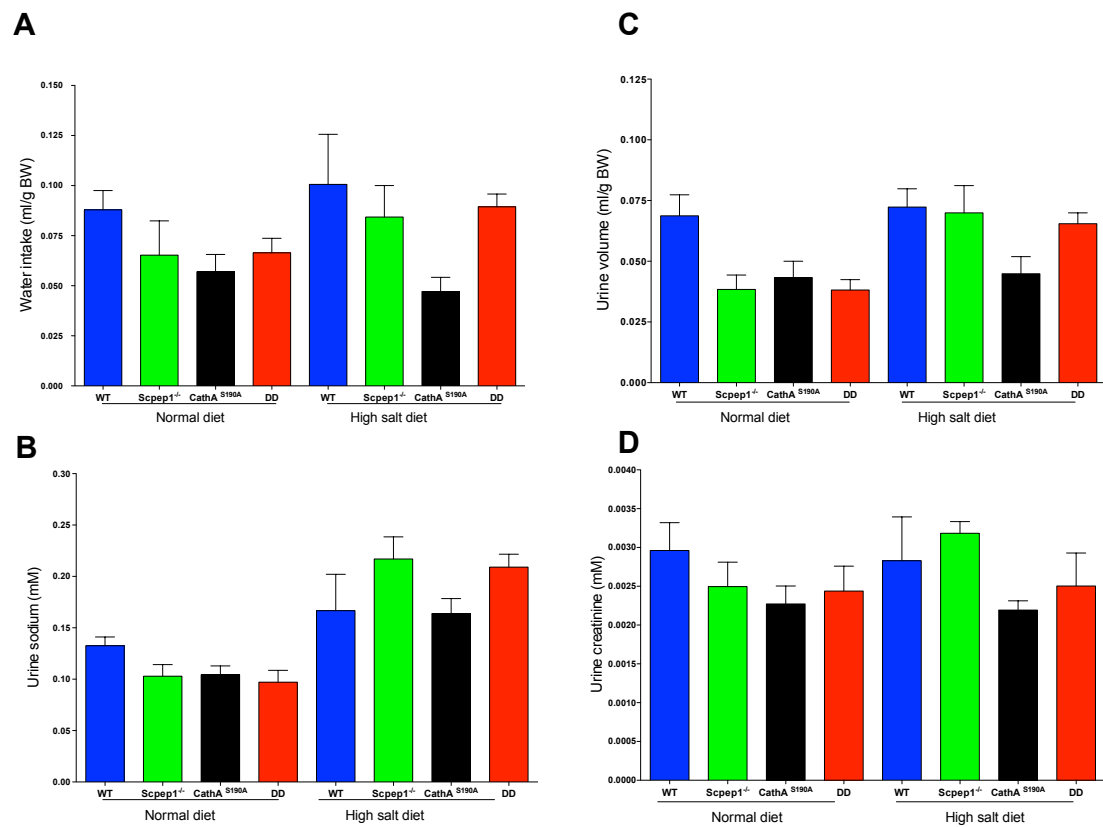

Supplementary figure 6

Supplement: Figure S6 — No significant differences in kidney function were observed between WT, Scpep1−/−, CathAS190A and double-deficient (DD) mice. Twenty four hour water intake was measured and urine collections obtained from 16 week-old male WT, Scpep1 −/−, CathAS190A and double-deficient (DD) mice fed with normal diet, or following two weeks on a high salt diet. Graphs present 24 h water intake (A) urine sodium (B), urine volume (C) and urine creatinine (D) measured as previously described. Values are shown as means (±S.E). N-value for each genotype is as follows: WT n = 5, DD n = 6, CathAS190A n = 6, Scpep1 −/− n = 7. (PDF) [file pgen.1004146.s006.pdf]

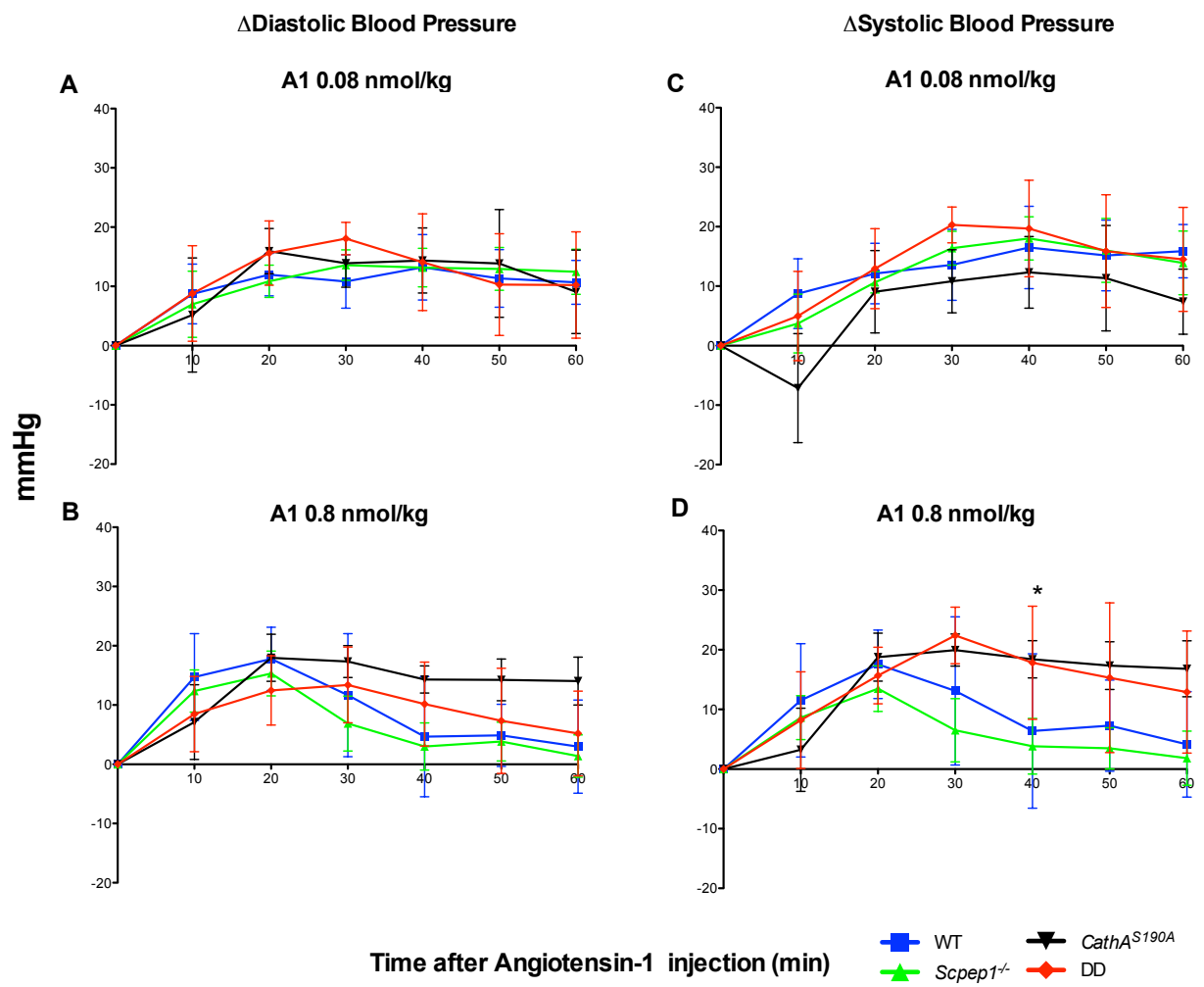

Supplementary figure 7

Supplement: Figure S7 — No significant difference was observed in diastolic (A, B) and systolic (C, D) blood pressure in response to systemic injections of AI. Sixteen week-old WT, Scpep1−/−, CathAS190A and double-deficient (DD) mice kept for two weeks on a high salt diet were intravenously injected with AI solution in saline (0.08 and 0.8 nmol/kg BW) or saline only. The pressure was recorded continuously every 2 min for 30 min before and one hour after injections. Changes in the blood pressure (ΔSP or ΔDP) were calculated as differences between the BP values recorded within 10 min intervals after the injections and the baseline BP values recorded within the 30 min interval before the injections. Two-way repeated measurements ANOVA was used to test differences between the mouse groups. N-value of each genotype is as follows: WT n = 5, DD n = 6, CathAS190A n = 6, Scpep1 −/− n = 7. (PDF) [file pgen.1004146.s007.pdf]

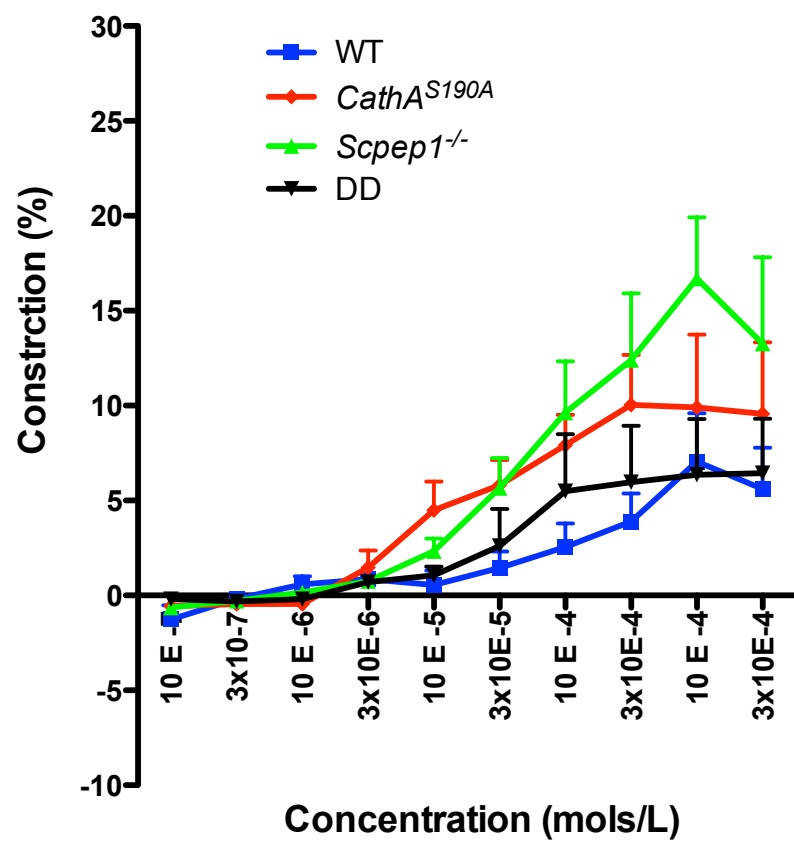

Supplementary figure 8

Supplement: Figure S8 — No significant difference was observed in the AI induced constriction of mesenteric arteries between CathAS190A, double deficient (DD) and WT mice. Mesenteric arteries isolated from sixteen week-old male WT, Scpep1 −/−, CathAS190A and double deficient mice were mounted onto glass capillaries in an artereograph chamber filled with cold oxygenated Krebs solution and treated with increasing concentrations of AI. Two-way repeated measurements ANOVA was used to test differences between the mouse groups. N-value of each genotype is as follows: WT n = 8, DD n = 8, CathAS190A n = 6, Scpep1 −/− n = 6. (PDF) [file pgen.1004146.s008.pdf]
